# Patients’ Experiences of the Transition to a 100% Single-Occupancy Patient Room Hospital in the Netherlands

**DOI:** 10.1177/19375867251381253

**Published:** 2025-10-23

**Authors:** Ralph Pruijsten, Elke de Groot-de Schepper, Annemarie J. B. M. de Vos, Erwin Ista, Liesbeth van Heel, Marianne J. E. van der Heijden, Monique van Dijk

**Affiliations:** 1 Section Nursing Science, Department of Internal Medicine, 6993 Erasmus University Medical Centre, Rotterdam, The Netherlands; 2 Department of Intensive Care, 36863 Ikazia Hospital, Rotterdam, The Netherlands; 3 Department of Cardiology, 89411 Amphia Hospital, Breda, The Netherlands; 4 Research Group for Continuing Professional Development in Nursing, 7898 Elisabeth-TweeSteden Hospital, Tilburg, The Netherlands; 5 Department of Public Health, Real Estate Department, 6993 Erasmus University Medical Centre, Rotterdam, The Netherlands

**Keywords:** single-occupancy rooms, patients’ satisfaction, hospital design, patients’ experiences

## Abstract

**Objectives:**

Our study examines experiences of patients admitted to multibedded patient rooms in a former hospital building, compared to patients’ experiences in single-occupancy patient rooms (SPRs) in a new hospital building, designed according to principles of a healing environment.

**Background:**

To improve patients’ privacy, comfort and infection control, newly built hospitals increasingly accommodate patients in SPRs.

**Methods:**

In a single-center, before-after study, patients completed a questionnaire of 40 items in four domains: privacy, sanitary facilities, patient room and ward layout. This substudy was embedded within the WELCOME study.

**Results:**

A total of 227 participants were involved in the before-measurement and 416 in the after-measurement. Patients considered the SPRs better in terms of privacy; more than 90% of participants (strongly) agreed with the privacy-related questionnaire items. Sanitary facilities, patient rooms and ward layout were also rated higher in the new hospital building. For most questionnaire items pertaining to these domains, more than 80% of patients in the new facility (strongly) agreed. 23.5% of respondents in the new building reported missing the companionship of fellow patients.

**Conclusions:**

Patients rated the 100% single-occupancy ward configuration more favorably than the former multi-occupancy layout, with enhanced privacy emerging as an important advantage. However, this same privacy can leave some patients feeling isolated. Future studies should explore targeted interventions—such as structured social activities or volunteer-led engagement—to mitigate loneliness and promote mobilization, while preserving the established benefits of SPRs.

## Background

SPRs are common practice in hospitals in the United States, since the implementation of the Guidelines for Design and Construction of Health Care Facilities in 2006 (American Institute of Architects, 2006, p. 347). Gradually, hospitals worldwide have begun to adopt this design, and today SPRs have become the norm in recently built hospitals in Western countries. SPRs are reported to improve patient well-being, reduce cross-infection rates and facilitate the work efficiency of health care workers (American Institute of Architects, 2006, p.347; Maben et al., 2016; Taylor et al., 2018; van der Schoor et al., 2022). Various studies have shown that SPRs contribute to patient satisfaction due to increased privacy, reduced noise levels and less disturbance from fellow patients (Larsen et al., 2014; Maben et al., 2016) and SPRs may have a favorable effect on sleep quality (Schafthuizen et al., 2023; Taylor et al., 2018). On the other hand, as described by Maben (Maben et al., 2015), possible downsides of SPRs include limited social interaction and risks of patient isolation.

Cusack and colleagues used semistructured interviews to explore patient experiences on surgical and medical wards in Australia, before and after a transition to a 100% SPR hospital (Cusack et al., 2023). Patients reported that SPRs markedly improved privacy, confidentiality, dignity, and overall comfort. An important advantage was having a private bathroom and toilet, which not only boosted convenience but also helped preserve patients’ privacy and sense of dignity. This was also described in a U.K. before-and-after study by Maben et al. (2016). Roughly two-thirds of patients favored SPRs, appreciating the increased comfort, sense of control, confidentiality, and flexible visiting. However, a minority mentioned feeling isolated. Whether these observations from Australia and the United Kingdom can readily be extrapolated to health-care settings elsewhere—such as the Netherlands—remains uncertain.

In May 2018, our hospital, a large University Medical Centre in the Netherlands, relocated to a new building with 100% SPRs. This transition aimed to reduce the number of hospital-acquired infections, to optimize the healing circumstances for patients and to increase occupancy rates. As described by Walker (2016), SPRs offer the best chance of maximum occupancy: by removing gender-mixing and cohorting constraints, 85 SPRs can match the capacity of 100 beds in a multibed setting, showing that a 100% SPR layout need not compromise overall bed numbers. To guide the design, the project adopted a healing-environment philosophy, which posits that elements such as privacy, low noise, abundant daylight and nature views, and patient control over light and temperature contribute to reduced stress, fewer complications and shorter lengths of stay (DuBose & Hadi, 2016; Huisman et al., 2012; Simonsen et al., 2022). Each SPR is therefore equipped with an ensuite bathroom, sound-absorbing finishes and a bedside tablet that allows patients to adjust lighting, blinds and climate settings. Glare-controlled daylight, curated artwork of biophilic views are incorporated to enhance visual comfort, while flexible visiting hours and daylight-filled family lounges promote social connection. Outside the patient rooms, simplified circulation routes shorten staff walking distances and reduce corridor noise, supporting a calm and efficient care environment. Collectively, these interdependent design strategies aim to convert architectural intent into measurable clinical, experiential and operational outcomes.

Supplemental File 1 contains drawings of the ward layouts of the former and new hospital buildings, and describes the healing environment elements incorporated into the new building.

In a recent survey study—conducted as a substudy embedded within the broader WELCOME project, which examines patients’ and staff perceptions, patient-safety issues and sleep quality in the new hospital building—we explored perceptions of our nursing staff of the transition to a 100% SPR hospital, designed according to principles of a healing environment. Respondents viewed the new layout mainly positive in terms of patient and ward environment (Pruijsten et al., 2024). However, they initially reported unfavorable effects on their own working conditions, collaboration, patient safety, and opportunities for patient monitoring in the new hospital building. One year later, these perceptions were reported less often in the new hospital building, which may be due to habituation to the new situation and reorganized work processes. Building on those staff-centered insights, the current study—also embedded as a WELCOME substudy—shifts the focus to patients. It aims to determine whether patients in our university hospital share the predominantly positive experiences reported in the United Kingdom (Maben et al., 2016) and Australia (Cusack et al., 2023), two countries whose health-care systems differ markedly from those in the Netherlands. Implementing 100% SPRs and healing-environment ward designs affects not only patient care but also overall health-care costs. In light of the existing literature and the contribution of our study, we observe that hospital managers worldwide frequently consult international evidence to inform decisions about the optimal balance between single- and multi-occupancy patient rooms.

Our research question is: to what extent do patients in a university hospital in the Netherlands appreciate the new hospital environment designed according to evidence-based healing environment principles?

We hypothesize that patients accommodated in the new 100% SPR hospital will report higher levels of overall satisfaction, perceived privacy, environmental control, and well-being than patients treated in the former multibed facility.

*Our research question is: to what extent do patients in a university hospital in the Netherlands appreciate the new hospital environment designed according to evidence-based healing environment principles?*

## Methods

### Design

The current study is embedded in a larger study (the WELCOME study) examining staff and patients’ perceptions, patients’ safety issues and sleep quality in the new hospital building. This single-center study had an uncontrolled before–after survey study design. The first data collection in the former hospital building took place from November 2017 through January 2018 (44 days) and the second in the new hospital building from November 2018 through January 2019 (63 days).

### Setting

In the former hospital building, the general wards accommodated patient rooms with two or four beds. The new hospital was designed according to evidence-based design principles to create a healing environment, with exclusively SPRs with an ensuite bathroom (Maben et al., 2016; Simonsen et al., 2022; Taylor et al., 2018; van Heel et al., 2024). Table 1 shows the differences in the rooms between the former and the new hospital building.

**Table 1.** Patient Room and Ward Environment Features in Former and New Hospital Building.

	Former hospital building	New hospital building
Number of patients in room	One, two, three, or four	One
Bathroom and toilet	In hallway (for max. 12 patients)	Ensuite bathroom
Room lighting control	Only bed light controlled by patient	All lighting controlled by patient (by patient tablet)
Temperature control in room	Not possible	Controlled to some extent by patient (by patient tablet)
Room doors		
‐Standard	Most with small window	Without window
‐Pressurized rooms	With windows in both doors	With window in both doors with controllable blinds
Room interior design	Standard, privacy curtain around the beds	Wooden door and façade, windows can be opened, soothing colors, orientation light under vertical bedhead panel; no privacy curtain
Sofa bed for rooming-in	No	Yes
Ceiling hoist	No	Yes
Nurse call system		
‐Patient (activating)	Button near bed and cord in bathroom	Button near bed and alarm on wristband
‐Nurse (receiver)	Light above door and alarm tone	Alarm on nurse's portable device
Lounge for self-service coffee and tea	No	Yes

### Participants

The study population comprised patients from 12 general wards, both surgical and medical. In the former hospital building, these wards were specialty-specific. After the relocation several were consolidated into so called care centers: organizational clusters that place patients with comparable nursing needs, but different diagnoses, on the same ward, thereby eliminating the former surgical-versus-medical divide. This restructuring did not alter existing ward policies or clinical procedures beyond the new ways of working required by the built environment, which applied equally across all wards. Moreover, a study by Korteland et al. (2025) found that the shift to a 100% SPR design had virtually no effect on the time nurses spent on individual tasks or on their multitasking levels compared to the former multibed setting. Convenience sampling was employed with the following inclusion criteria: age ≥ 18 years, ability to communicate verbally in Dutch, and admission for at least two consecutive nights before enrollment. Patients with documented cognitive impairment were excluded. During each data-collection period, every weekday all patients meeting these criteria were invited to participate.

### Materials

The questionnaire administered to each patient consisted of three sections: demographic data, the EuroQol five-dimensional questionnaire (EQ-5D-3L) and the ward environment questionnaire.

#### Background Characteristics of Participants

Age, sex, educational level, retirement status and number of fellow patients in the room were collected as background data.

#### Health-Related Quality of Life

We selected the Dutch EQ-5D-3L, together with the EuroQol visual analogue scale (EQ-VAS), to assess health-related quality of life in both patient cohorts. These instruments are brief and diagnosis-independent. The EQ-5D-3L, in particular, has demonstrated strong reliability and validity in large Dutch and international patient samples (Mulhern et al., 2018; Selivanova et al., 2018; Szende et al., 2014; Vartiainen et al., 2017; Xie et al., 2014). The generic design of both instruments makes it possible to compare diverse patient groups—medical and surgical—between the former and new hospital building. The EQ-5D-3L captures five categories—mobility, self-care, usual activities, pain/discomfort and anxiety/depression—each rated at three levels (“no,” “some,” or “extreme” problems). This results in a health status index score ranging from −0.329 (worst possible health state) to 1 (perfect health state) according the Dutch EQ-5D tariff (Versteegh et al., 2016). The EQ-VAS was used to rate participants’ self-perceived health at the day of assessment, with anchors 0 “worst imaginable health state” and 100 “best imaginable health state” (Evans et al., 2018).

#### Ward Environment Questionnaire

Our “Ward Environment” questionnaire was based on a questionnaire designed by Maben et al. (2015). With permission, we modified the content to make it suitable for a patient questionnaire and included elements characterizing this new hospital's ward environment. Although the questionnaire was adapted, it was not pilot-tested before use, and the version employed in this study had not been used by other researchers. The final questionnaire contained 40 closed items and used a five-point Likert scale ranging from “totally disagree” to “totally agree.” In addition, the option “not applicable” was provided. The items concerned four main features: privacy, bathroom facilities, room design and ward-layout. In addition, three open questions aim to clarify topics such as rooming-in and benefits of both SPRs and multioccupancy rooms. The final item invited participants to suggest which features may add to the room's comfort. To evaluate the risk of loneliness, the item “I do miss a fellow patient to talk to” was added to the questionnaire administered in the new hospital building. The Ward Environment Questionnaire is shown as Supplemental File 2.

### Data Collection Methods

Research assistants were trained by a research nurse in informed consent procedures, patient approach, and hygiene protocols. Following a supervised trial period, they were permitted to work independently. To distinguish them from ward staff, they wore shirts bearing the WELCOME logo. Eligible patients were identified by nursing staff. Each patient received a clear explanation of the study and was informed that participation was voluntary and could be declined or discontinued at any time. Written informed consent was obtained prior to participation. Patients then completed the online questionnaire on a tablet; if needed, the assistant read the questions aloud and recorded their responses verbatim.

### Data Analysis

Descriptive statistics were used to summarize the characteristics of the data set. The Kolmogorov–Smirnov test was used to establish whether continuous data were normally distributed. Normally distributed variables are presented as mean (standard deviation), nonnormally distributed variables as median (interquartile range). The independent-samples t-test or the Mann–Whitney-U-test, whichever was appropriate, was used to compare continuous variables across the two measurement periods. Chi-square (χ²) tests compared categorical variables between the two periods. In addition, sex differences in the proportion of patients who reported missing a fellow patient were examined with a χ² test, while age differences between patients who did versus did not miss a fellow patient were assessed with a Mann–Whitney U-test.

The R-package “EQ5D” was used to calculate the EQ-5D utility index in order to determine the comparability of the two patient samples in the former and new hospital building (Morton and Nijjar, 2021). We calculated the index by selecting the EQ-5D-3L, type TTO, the country-specific value set (the Netherlands) and “multiple calculations.” To improve interpretability of the data, the five-point Likert scale response categories were collapsed into three response categories, namely “totally agree and agree,” “not agree, not disagree,” and “totally disagree and disagree.” A *p*-value of less than .05 was considered to reflect statistical significance. Analyses were performed in IBM Statistical Package of the Social Sciences for Windows, version 19 (IBM Corp., Armonk, N.Y., USA) and in RStudio Team (2016) (RStudio, Inc., Boston, MA).

### Ethical Considerations

All participants provided written informed consent. The Erasmus MC Medical Ethics Review Board approved the study (MEC 2017-1103).

## Results

Two hundred and twenty-seven participants were involved in the before-measurement versus 416 in the after-measurement. The participants in the after measurement were older, with a median age of 62 versus 60 years (*p* = .029). No significant differences were found in sex and proportion of retirees. In the former hospital building, 28.6% of the patients were the only occupant of the (multioccupancy) room, as opposed to 100% of the patients in the new hospital building. The median admission duration of patients at the time of the assessment was three days in both hospital buildings (interquartile range (IQR): 1–8 days in the former hospital building, 1–6 days in the new hospital building; *p* = .078). The patient characteristics are shown in Table 2.

**Table 2.** Patient Characteristics.

Characteristics	Former hospital building (*N* = 227)	New hospital building (*N* = 416)	*p*-value
Age, median (IQR), y	60 (45–70)	62 (53–70)	.029
*n*	227	415	
Sex: female, n (%)	103 (45.4)	192 (46.2)	.850
*n*	227	416	
Retired, *n* (%)	74 (38.3)	165 (39.7)	.756
*n*	193	416	
Number of fellow patients in the room, *n* (%)			
‐None	57 (28.6)	416 (100%)	
‐One	55 (27.7)		
‐Two	30 (15.1)		
‐Three	57 (28.6)		
*n*	199		
Hospital stay in days at assessment; median (IQR)	3 (1–8)	3 (1–6)	.078
*n*	227	416	

*Note.* IQR: interquartile range.

### Health-Related Quality of Life

As shown in Table 3, both the self-reported health state and the overall EQ-5D scores of the participants in the former and new hospital building did not differ statistically significantly, with the exception of “Usual Activities” and “Anxiety/Depression.” In the new hospital building, a larger proportion of participants (56.7%) reported no problems with “Usual Activities,” compared to the former hospital building (44.4%). The proportion of bedridden participants was less than 10% in both hospital buildings (respectively 8.4% and 5.6%).

**Table 3.** Patients’ Health-Related Quality of Life.

Characteristics	Former hospital building (*N* = 227)	New hospital building (*N* = 416)	*p*-value
Mobility, *n* (%)			
‐No problems with walking	118 (55.1)	244 (59.1)	.338
‐Some problems with walking	78 (36.4)	146 (35.4)	
‐Confined to bed	18 (8.4)	23 (5.6)	
*n*	214	413	
Self-care, *n* (%)			
‐No problems with self-care	151 (70.6)	280 (67.6)	.465
‐Some problems with washing or dressing	50 (23.4)	114 (27.5)	
‐Unable to wash or dress	13 (6.1)	20 (4.8)	
*n*	214	414	
Usual activities, *n* (%)			
‐No problems with performing usual activities	95 (44.4)	234 (56.7)	<.001
‐Some problems with performing usual activities	72 (33.6)	146 (35.4)	
‐Unable to perform usual activities	47 (22.0)	33 (8.0)	
*n*	214	413	
Pain/discomfort, *n* (%)			
‐No pain or discomfort	103 (48.1)	223 (53.9)	.379
‐Some pain or discomfort	91 (42.5)	159 (38.4)	
‐Extreme pain or discomfort	20 (9.3)	32 (7.7)	
*n*	214	414	
Anxiety/depression, *n* (%)			
‐Not anxious or depressed	156 (72.9)	279 (67.7)	.003
‐Moderately anxious or depressed	47 (22.0)	127 (30.8)	
‐Extremely anxious or depressed	11 (5.1)	6 (1.5)	
*n*	214	412	
Self-reported health status index score (EQ-VAS);			
median (interquartile range)	60.5 (50–75)	65.0 (50–76)	.262
*n*	208	411	
EQ-5D Values; median (interquartile range)	0.78 (0.53–0.89)	0.80 (0.64–1.00)	.060
*N*	227	416	

*Note.* IQR- interquartile range.

### Privacy

**Figure 1.**
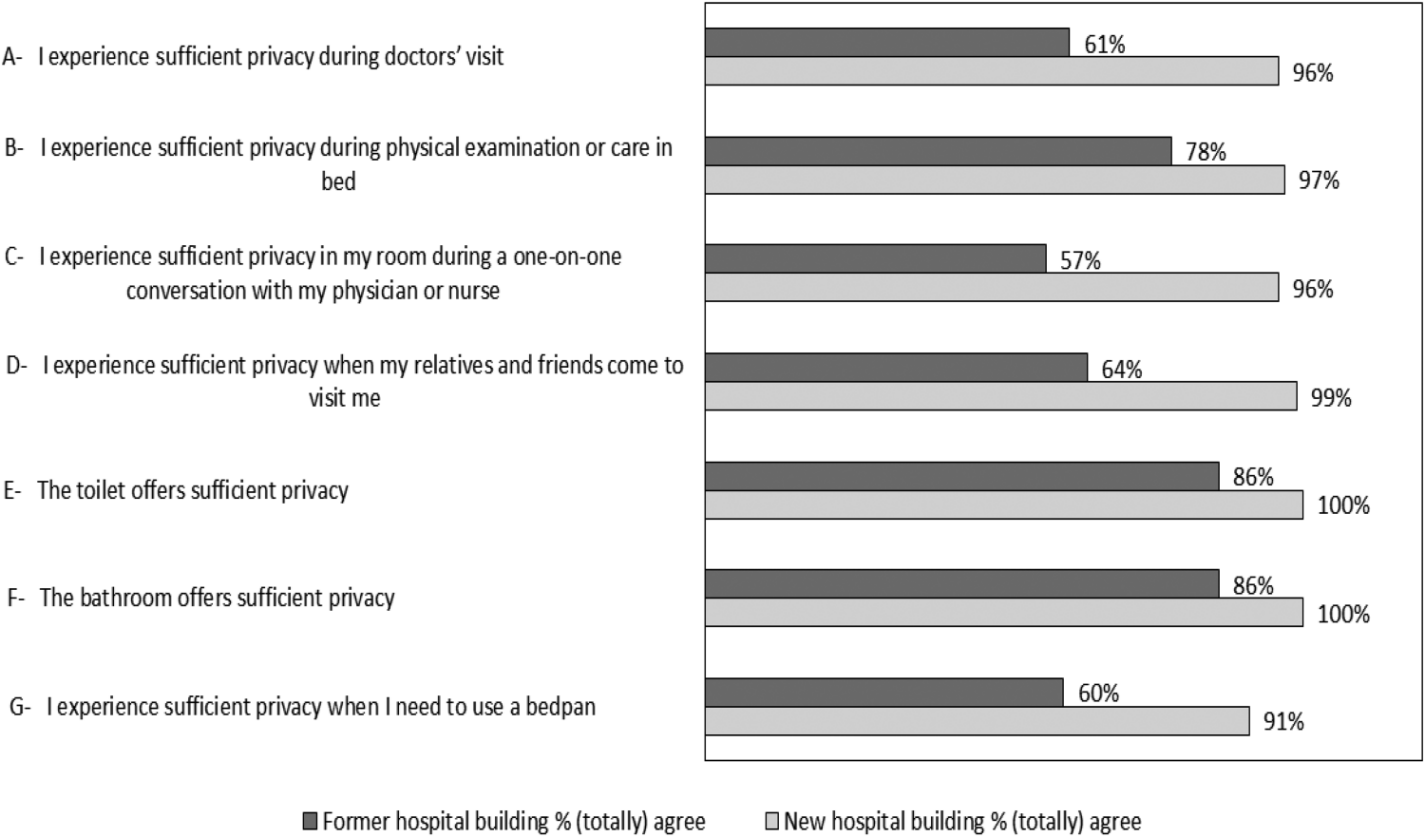
Privacy Items Depicted in Percentages for the Former and New Hospital Building.

*Note.* Proportions of Participants Who (Totally) Agreed with the Statements Concerning Privacy, for Former and New Hospital Building (*p*-Value for All Statements <.05).

Figure 1 provides the percentages of participants who (totally) agreed with the statements concerning privacy, in the former and new hospital buildings. Overall, we saw an increase in appreciation of the privacy in the new hospital building compared to the former hospital building.

In particular, we found an improvement in appreciation of privacy during conversations with medical or nursing staff (items A and C), visits from relatives and friends (item D) and when using a bedpan (item G). The detailed responses on the Likert scales on privacy are shown in Supplemental File 3.

### Sanitary Facilities

**Figure 2.**
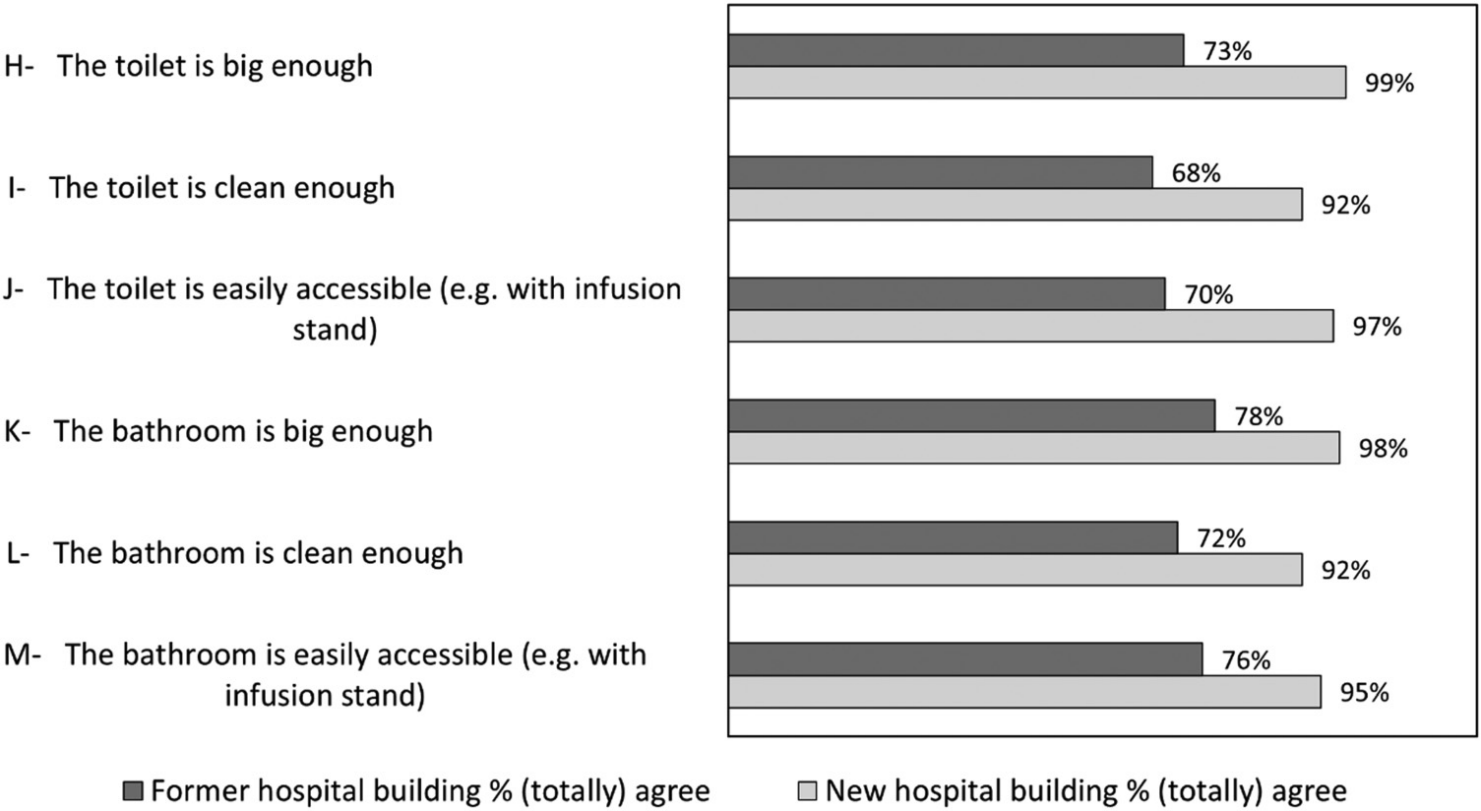
Sanitary Facilities Items Depicted in Percentages for the Former and New Hospital Building.

*Note.* Proportions of Participants Who (Totally) Agreed with the Statements Concerning Sanitary Facilities, for Former and New Hospital Building (*p*-Value for All Statements <0.05).

Figure 2 shows the percentages of participants who (totally) agreed with the statements concerning sanitary facilities, broken down for the former and new hospital building. Regarding all items, we saw an increase in appreciation of the sanitary facilities in the new hospital building compared to the former hospital building. All data concerning responses on Sanitary facilities are shown in Supplemental File 4.

### Room Design

**Figure 3.**
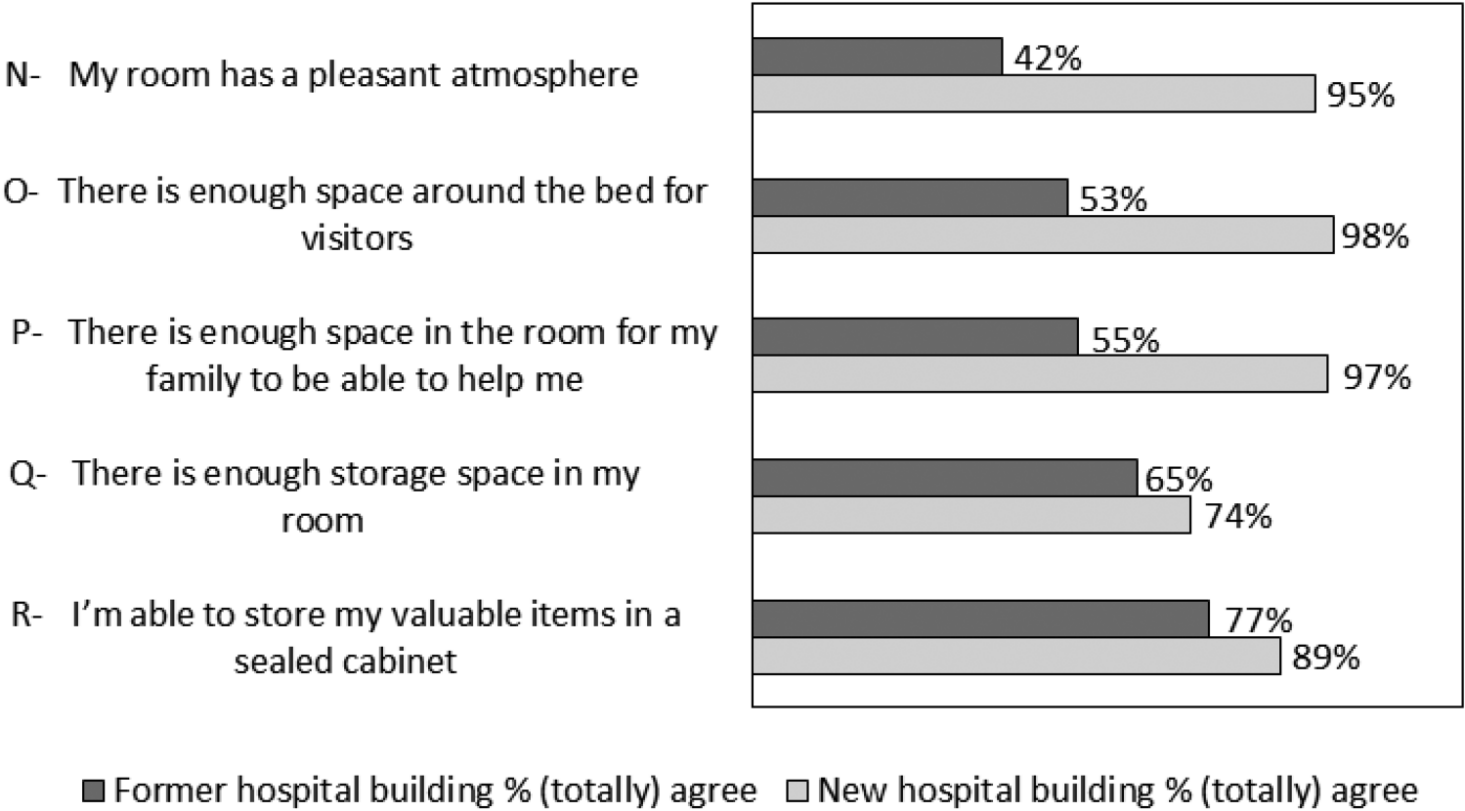
Room Design Items Depicted in Percentages for the Former and New Hospital Building.

*Note.* Proportions of Participants Who (Totally) Agreed with the Statements Concerning Room Design, for Former and New Hospital Building (p-Value for All Statements <.05).

Figure 3 shows the percentages of participants who (totally) agreed with the statements concerning room design, for the former and new hospital building. On all items there was an improvement comparing the former hospital building with the new hospital building. In Supplemental File 5, all data are shown concerning responses on Room design.

### Ward lay-out

**Figure 4.**
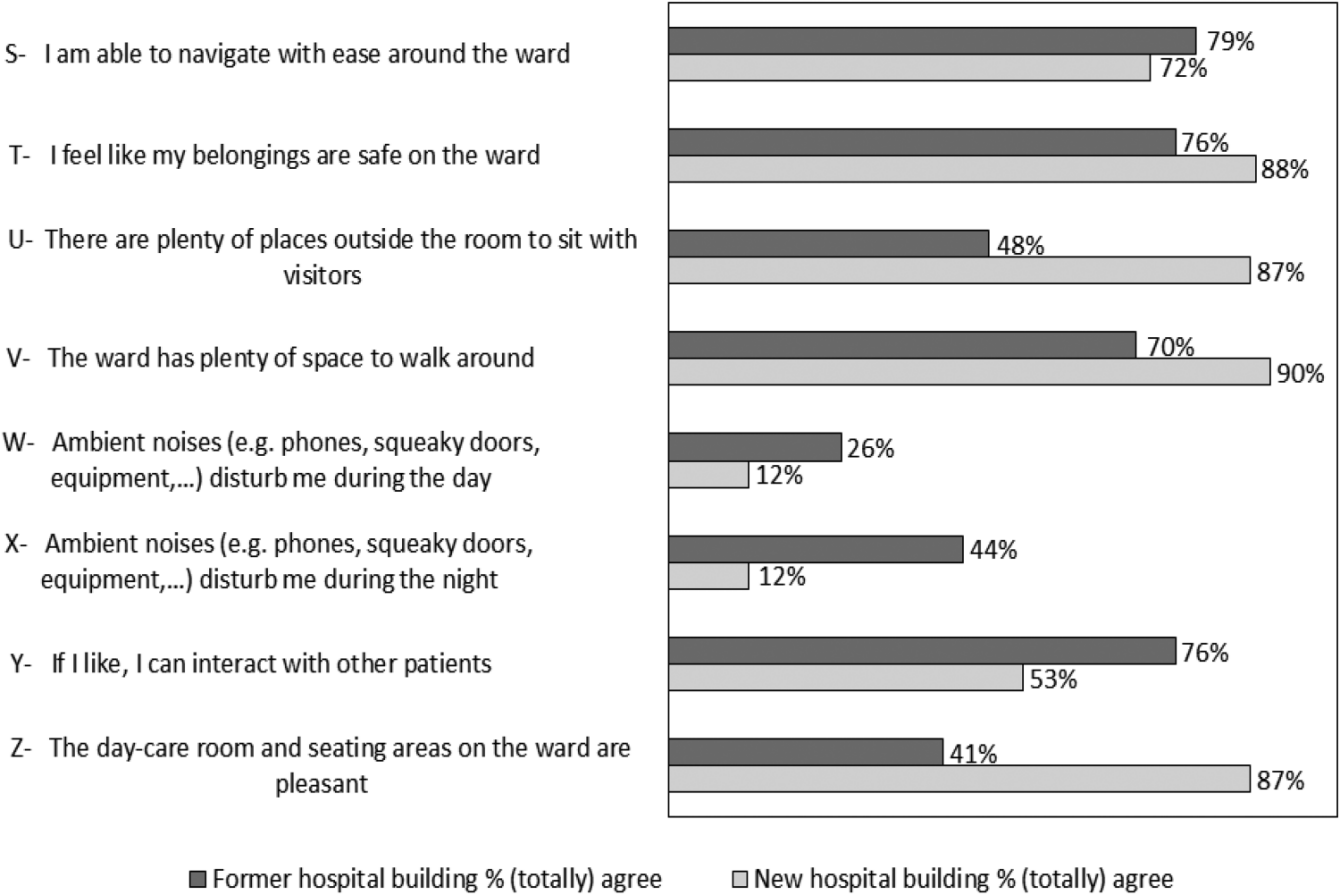
Ward Lay-out Items Depicted in Percentages for the Former and New Hospital Building.

*Note*. Proportions of Participants Who (Totally) Agreed with the Statements Concerning ward Lay-out, for Former and New Hospital Building (*p*-Value for All Statements <.05).

The percentages of participants who (totally) agreed with the statements concerning ward lay-out in former and new hospital building are shown in Figure 4. Interacting with other patients was considered feasible in the former hospital building by 76% of the patients opposed to 53% of patients in the new hospital building. In line with this: almost a quarter of the participants (92 out of 391 patients: 23.5%) in the new hospital indicated that they sometimes missed contact with fellow patients in their room. This applied to 21.0% of male and 26.5% of female patients (*p* = .20). Patients who reported missing contact tended to be somewhat older (median age 66 years, IQR 57–71) than those who did not (median 62 years, IQR 51–70), (*p* = .056).

In the new hospital building, more participants are positive about the opportunities to walk around the ward and sit with visitors, than in the former hospital building. In the former hospital building, almost half of the participants (44%) reported being bothered by ambient noise during the night and a quarter of the participants (26%) during the day. This was significantly less in the new hospital building and applied to 12% during the night and day (*p* < .001). All data concerning responses on Ward lay-out are shown in Supplemental File 6.

## Discussion

In this uncontrolled before-after survey study, we investigated the experiences of patients admitted to our former hospital building with multi-bedded rooms compared to those of patients admitted to a newly built hospital with 100% single-occupancy rooms. Patients in the new hospital building were generally very satisfied with the 100% SPRs layout and perceived room design, privacy, sanitary facilities and most aspects of the ward layout better in the new hospital building than in the former hospital building with mainly two- and four-bedded rooms. Our findings confirm those of Maben et al. and Cusack et al. (Cusack et al., 2023; Maben et al., 2016). Table 4 provides a side-by-side comparison of our patient-reported experiences alongside theirs. In terms of patient mix our study is closest to the Australian work by Cusack and colleagues, which focused exclusively on adult medical-surgical wards. The U.K. evaluation by Maben et al., by contrast, included four different settings—an acute-admissions unit, a geriatrics ward and a maternity ward in addition to a general surgical ward—so any comparisons with their findings must be interpreted in light of these extra obstetric and frail-elderly patient groups. Methodologically our approach diverges from both earlier studies: we used patient questionnaires, while they relied primarily on interviews (plus staff questionnaires). This choice allowed us to recruit a substantially larger patient sample and to incorporate validated measures of health-related quality of life (EQ-5D-3L and EQ-VAS), instruments that neither Maben nor Cusack applied.

*Patients in the new hospital building were generally very satisfied with the 100% SPRs layout and perceived room design, privacy, sanitary facilities and most aspects of the ward layout better in the new hospital building than in the former hospital building with mainly two- and four-bedded rooms.*

**Table 4.** Comparison of Patient-Reported Experiences With 100% Single-Occupancy Patient Rooms in Three Before–After Hospital Studies (Australia, UK, the Netherlands).

Theme	Cusack et al. (2023)—Australia	Maben et al. (2016)—United Kingdom	Present study—the Netherlands
Study design and context	Before–after mixed-methods study in one tertiary hospital: Stage 1 (2016) premove versus Stage 2 (2021) five years after relocation to 100% SPRs	Mixed-methods pre/postmove comparison of a new NHS hospital with 100% SPRs, including two control hospitals	Uncontrolled before-after survey in one hospital: baseline in former hospital building with mostly 2-/4- bedded wards versus follow-up in new 100% SPR building
Patient data-collection method	Semistructured interviews (*n* = 12 premove, *n* = 12 postmove)	pre- and post-semistructured interviews (*n* = 32)	Tablet questionnaire, self-completed; assistant entered answers if needed (*n* = 227 pre; *n* = 416 post)
Participants (ward types)	Four adult medical and surgical wards	Medical assessment unit, surgical, medical (older-people) and maternity wards	Twelve general wards (surgical and medical) reorganized into mixed *care-center* units after the move
Key *positive* experiences	Privacy and dignity; Quieter, Better sleep; En-suite comfort; Better family visiting	Privacy and control; Flexible visiting; Comfort/“feeling at home”	More privacy improved communication with staff en-suite facilities better room/ward environment
Key *negative* experiences	Minority felt isolated	∼ ⅓ reported loneliness; Men more likely to prefer multibedded rooms	Feelings of isolation in 24% of patients
Preference for room type	Majority favored SPRs; 2/12 patients (≈ 17%) preferred multibedded rooms for company	∼ ⅔ preferred SPRs	Clear preference for SPRs over former multioccupancy layout

*Note.* SPR: single-occupancy patient room.

Staff questionnaires were included in the Cusack and Maben studies, only patient-reported findings are presented here.

Our findings on privacy are consistent with those of Devlin et al., who described the importance of privacy of both room and bathroom for hospitalized patients (Devlin et al., 2016). This is also supported by a systematic review from 2023 (Bertuzzi et al., 2023). In a scoping review, Søndergaard et al. (2022) conclude that patients staying in SPRs feel empowered when it comes to their privacy, in terms of being able to close the door and the presence of an ensuite bathroom. In our study, patients in SPRs benefit from privacy and less disturbance. Privacy during communication between healthcare professionals and patients was also perceived better in the new hospital building. It is highly likely that patients in a multi-occupancy room -realizing that fellow patients can overhear conversations- may withhold information from healthcare professionals (Larsen et al., 2014) or not feel able to discuss their condition and concerns as much (Maben et al., 2016).

Almost all participants found the atmosphere of the room pleasant in the new hospital building. From a patient perspective the room and the facilities with its increased comfort were reported to provide a hotel room experience given the perceived comfort supporting findings from other studies (Maben et al., 2016; Søndergaard et al., 2022). A downside might be that patients tend to stay in their room, are more sedentary as we noted in a study evaluating the mobility of patients in our SPRs (Schafthuizen et al., 2024). The reduction of noise was a major improvement in the new hospital building. Noise was perceived as much less annoying, especially during the night. The absence of fellow patients and the possibility to close the bedroom door—and thus reduce noise from outside—is conducive to good sleep (Søndergaard et al., 2022). Noise from other patients or staff is a main reason for disturbance during the night (Adams et al., 2024; Cusack et al., 2023; Dobing et al., 2016; Garside et al.; 2018). However, in contrast to findings of Adams et al., Cusack et al., and Dobing et al., in a study in our hospital we found only a slightly better self-reported sleep quality for patients in SPRs than for patients in multioccupancy rooms (Schafthuizen et al., 2023). This may be because sleep quality is affected not only by noise levels, but also by illness-related factors such as nausea and pain, disruption of the day-night rhythm, toilet visits and also early awakenings by healthcare workers (Schafthuizen et al., 2023).

*The reduction of noise was a major improvement in the new hospital building.*

A possible downside of SPRs is that patients may feel lonely because there is less interaction with other patients. The planners and designers of our new hospital building had anticipated that patients would make use of lounge areas to meet fellow patients or visitors. However, although 87% of our surveyed patients rated the daycare room and seating areas as pleasant, it appeared that these lounge areas were hardly visited. Comparable underutilization of lounge areas has been reported in other European hospitals (Kevdzija et al., 2022; Ribbe Kelso et al., 2023). Greater proximity to patient rooms and programed activities may be needed to convert perceived appeal into actual uptake. However, in our new hospital building, areas with a coffee machine closer to the rooms, meant to invite patients to step out of their rooms, were sometimes repurposed as break rooms for nurses. This was also reported by Maben et al. (2016), with patients not sure where the patients lounge room was or whether they were allowed to visit. Feelings of social isolation and loneliness in SPRs are well documented. A scoping review by Søndergaard et al. (2022) identified nine studies in which patients reported isolation, loneliness and a desire for company, while Maben et al. (2016) found that patients in SPRs could feel isolated because task-oriented staff had little time for conversation. We noticed similar results in our study: although most patients were satisfied with their single room, almost one-quarter nevertheless experienced occasional loneliness, despite extended visiting hours and rooming-in facilities. We observed no sex differences, in contrast with Maben et al. (2016), who noted that nearly half of male patients preferred multibed accommodation. Recent studies likewise show that some older patients value companionship more than the extra privacy of an SPR (Bertuzzi et al., 2023; Cusack et al., 2023). Among our patients, the wish for contact was not statistically significantly associated with age, which may partly be explained by the fact that the median length of stay at the time of assessment was only three days (see Table 2). It could be interesting to consider involving volunteers to encourage more interaction between patients or at least enhance the use of the lounge areas, by orientating new patients not only to their own room but to the whole ward including the lounge.

### Strengths and Limitations

Our study was conducted in a large university medical center, which provided the opportunity to survey a broad population with a wide range of ages, health states and lengths of stay. Still, several limitations need to be addressed. First, this is a single-center study, which may have created bias due the specific patient population in a university hospital. We aimed to overcome this limitation by including patients from 12 wards with different medical and surgical specialities. Nevertheless, a multicenter study would provide a broader insight for this subject. Second, the study has a before-after design without a control group, which limits the strength of the conclusions. Third, a limitation of our study is that the adapted ward-environment questionnaire has not yet been formally validated; further reliability and validity testing is therefore required. Fourth, it is challenging to distinguish whether patients’ perceived improvements are due to the introduction of single patient rooms or simply the result of being in a new, modern environment. The comparison between an old, poorly maintained building and a brand-new building should also be taken into account, in addition to the shift from multiple- to single-occupancy patient rooms. Fifth, we were unable to compare wards in the former hospital building with wards in the new building, because the new hospital building is in part set up with care centers instead of more traditional surgical or medical wards. Comparing wards could have provided us with more specific information and differences in experience between patients admitted on a surgical or medical ward. Sixth, this study is a survey study. Therefore, we might have missed relevant information which could come to the surface during in-depth interviews. Relocation to a new hospital comes with many more changes, such as work processes and facilities. We are currently studying these topics.

## Conclusions

Our patients rated the 100% single-occupancy ward configuration more favorably than the former multioccupancy layout. Benefits include more privacy, improved en-suite sanitary facilities, an overall better room and ward environment, and improved communication with healthcare professionals. However, the added privacy can leave some patients feeling isolated -despite visiting hours being extended in the new facility and rooming-in facilities. Future studies should explore targeted interventions—such as structured social activities or volunteer-led engagement—to mitigate loneliness and promote mobilization, while preserving the established benefits of SPRs.

*The added privacy can leave some patients feeling isolated -despite visiting hours being extended in the new facility and rooming-in facilities.*

## Implications for Practice

- Patients prefer SPRs with ensuite bathrooms over multibed rooms; they provide greater privacy and an overall better room environment.
- SPR wards reduce the ambient noise patients perceive -both during the day and at night.
- In SPRs, some patients may feel lonely, despite extended visiting hours and rooming-in facilities.

## Supplemental Material

sj-docx-1-her-10.1177_19375867251381253 - Supplemental material for Patients’ Experiences of the Transition to a 100% Single-Occupancy Patient Room Hospital in the Netherlands Supplemental material, sj-docx-1-her-10.1177_19375867251381253 for Patients’ Experiences of the Transition to a 100% Single-Occupancy Patient Room Hospital in the Netherlands by Ralph Pruijsten, Elke de Groot-de Schepper, Annemarie J. B. M. de Vos, Erwin Ista, Liesbeth van Heel, Marianne J. E. van der Heijden and Monique van Dijk in HERD: Health Environments Research & Design Journal

sj-jpg-2-her-10.1177_19375867251381253 - Supplemental material for Patients’ Experiences of the Transition to a 100% Single-Occupancy Patient Room Hospital in the Netherlands Supplemental material, sj-jpg-2-her-10.1177_19375867251381253 for Patients’ Experiences of the Transition to a 100% Single-Occupancy Patient Room Hospital in the Netherlands by Ralph Pruijsten, Elke de Groot-de Schepper, Annemarie J. B. M. de Vos, Erwin Ista, Liesbeth van Heel, Marianne J. E. van der Heijden and Monique van Dijk in HERD: Health Environments Research & Design Journal

sj-jpg-3-her-10.1177_19375867251381253 - Supplemental material for Patients’ Experiences of the Transition to a 100% Single-Occupancy Patient Room Hospital in the Netherlands Supplemental material, sj-jpg-3-her-10.1177_19375867251381253 for Patients’ Experiences of the Transition to a 100% Single-Occupancy Patient Room Hospital in the Netherlands by Ralph Pruijsten, Elke de Groot-de Schepper, Annemarie J. B. M. de Vos, Erwin Ista, Liesbeth van Heel, Marianne J. E. van der Heijden and Monique van Dijk in HERD: Health Environments Research & Design Journal

sj-jpg-4-her-10.1177_19375867251381253 - Supplemental material for Patients’ Experiences of the Transition to a 100% Single-Occupancy Patient Room Hospital in the Netherlands Supplemental material, sj-jpg-4-her-10.1177_19375867251381253 for Patients’ Experiences of the Transition to a 100% Single-Occupancy Patient Room Hospital in the Netherlands by Ralph Pruijsten, Elke de Groot-de Schepper, Annemarie J. B. M. de Vos, Erwin Ista, Liesbeth van Heel, Marianne J. E. van der Heijden and Monique van Dijk in HERD: Health Environments Research & Design Journal

sj-jpg-5-her-10.1177_19375867251381253 - Supplemental material for Patients’ Experiences of the Transition to a 100% Single-Occupancy Patient Room Hospital in the Netherlands Supplemental material, sj-jpg-5-her-10.1177_19375867251381253 for Patients’ Experiences of the Transition to a 100% Single-Occupancy Patient Room Hospital in the Netherlands by Ralph Pruijsten, Elke de Groot-de Schepper, Annemarie J. B. M. de Vos, Erwin Ista, Liesbeth van Heel, Marianne J. E. van der Heijden and Monique van Dijk in HERD: Health Environments Research & Design Journal

sj-jpg-6-her-10.1177_19375867251381253 - Supplemental material for Patients’ Experiences of the Transition to a 100% Single-Occupancy Patient Room Hospital in the Netherlands Supplemental material, sj-jpg-6-her-10.1177_19375867251381253 for Patients’ Experiences of the Transition to a 100% Single-Occupancy Patient Room Hospital in the Netherlands by Ralph Pruijsten, Elke de Groot-de Schepper, Annemarie J. B. M. de Vos, Erwin Ista, Liesbeth van Heel, Marianne J. E. van der Heijden and Monique van Dijk in HERD: Health Environments Research & Design Journal

sj-docx-7-her-10.1177_19375867251381253 - Supplemental material for Patients’ Experiences of the Transition to a 100% Single-Occupancy Patient Room Hospital in the Netherlands Supplemental material, sj-docx-7-her-10.1177_19375867251381253 for Patients’ Experiences of the Transition to a 100% Single-Occupancy Patient Room Hospital in the Netherlands by Ralph Pruijsten, Elke de Groot-de Schepper, Annemarie J. B. M. de Vos, Erwin Ista, Liesbeth van Heel, Marianne J. E. van der Heijden and Monique van Dijk in HERD: Health Environments Research & Design Journal

sj-docx-8-her-10.1177_19375867251381253 - Supplemental material for Patients’ Experiences of the Transition to a 100% Single-Occupancy Patient Room Hospital in the Netherlands Supplemental material, sj-docx-8-her-10.1177_19375867251381253 for Patients’ Experiences of the Transition to a 100% Single-Occupancy Patient Room Hospital in the Netherlands by Ralph Pruijsten, Elke de Groot-de Schepper, Annemarie J. B. M. de Vos, Erwin Ista, Liesbeth van Heel, Marianne J. E. van der Heijden and Monique van Dijk in HERD: Health Environments Research & Design Journal

sj-docx-9-her-10.1177_19375867251381253 - Supplemental material for Patients’ Experiences of the Transition to a 100% Single-Occupancy Patient Room Hospital in the Netherlands Supplemental material, sj-docx-9-her-10.1177_19375867251381253 for Patients’ Experiences of the Transition to a 100% Single-Occupancy Patient Room Hospital in the Netherlands by Ralph Pruijsten, Elke de Groot-de Schepper, Annemarie J. B. M. de Vos, Erwin Ista, Liesbeth van Heel, Marianne J. E. van der Heijden and Monique van Dijk in HERD: Health Environments Research & Design Journal

sj-docx-10-her-10.1177_19375867251381253 - Supplemental material for Patients’ Experiences of the Transition to a 100% Single-Occupancy Patient Room Hospital in the Netherlands Supplemental material, sj-docx-10-her-10.1177_19375867251381253 for Patients’ Experiences of the Transition to a 100% Single-Occupancy Patient Room Hospital in the Netherlands by Ralph Pruijsten, Elke de Groot-de Schepper, Annemarie J. B. M. de Vos, Erwin Ista, Liesbeth van Heel, Marianne J. E. van der Heijden and Monique van Dijk in HERD: Health Environments Research & Design Journal

sj-docx-11-her-10.1177_19375867251381253 - Supplemental material for Patients’ Experiences of the Transition to a 100% Single-Occupancy Patient Room Hospital in the Netherlands Supplemental material, sj-docx-11-her-10.1177_19375867251381253 for Patients’ Experiences of the Transition to a 100% Single-Occupancy Patient Room Hospital in the Netherlands by Ralph Pruijsten, Elke de Groot-de Schepper, Annemarie J. B. M. de Vos, Erwin Ista, Liesbeth van Heel, Marianne J. E. van der Heijden and Monique van Dijk in HERD: Health Environments Research & Design Journal

## Abbreviations

EQ-5D-3L: EuroQol five-dimensional questionnaire
EQ-VAS: EuroQol Visual Analogue Scale
HRQoL: health-related quality of life
IQR: interquartile range
SPR: single-occupancy room
UMC: University Medical Centre
