# Supplementary material for: Patients’ Experiences of the Transition to a 100% Single-Occupancy Patient Room Hospital in the Netherlands
Source: HERD. 2025 Oct 23;19(1):184–98. doi: 10.1177/19375867251381253 (PMC12715026; doi:10.1177/19375867251381253)
Supplement: sj-docx-1-her-10.1177_19375867251381253 - Supplemental material for Patients’ Experiences of the Transition to a 100% Single-Occupancy Patient Room Hospital in the Netherlands [file sj-docx-1-her-10.1177_19375867251381253.docx]

The wards in the former hospital building featured a double-corridor layout, with centrally located storage areas, kitchen, and other support facilities. In contrast, the new wards adopted the bed-cluster (‘sengetun’) set-up, with 3-4 clusters of 8 SPRs forming one ward. The bed-clusters are monitored from decentralized nursing stations in an open setting. Each ward also includes a centralized on-ward station, located close to the medication room, dirty utility, pneumatic tube station, and the ward secretary’s workplace. This central station supports ward operations and serves to bring staff together during evening and night shifts (see Photo 1). Near the decentralized nursing stations, trolleys (mobile cupboards) provide a 24-hour supply of nursing materials and linen. This arrangement reduces walking distances for nurses when retrieving frequently used items, which are no longer stocked in patient rooms to prevent waste in the event of room disinfection. The trolleys are restocked daily by facility care workers from more centrally located storage areas, which are easily accessible to logistics staff. These features -decentralized nursing stations and trolleys- were implemented to address staff concerns about increased walking distances due to the greater façade length required for SPRs. Working with substations and smaller, dedicated teams was intended to increase opportunities for patient interaction. The effects of these measures have recently been reported in a published study (Korteland et al. ,2025)

Early in the project, the design team developed ‘design notices’ on topics such as indoor climate, greenery, visibility, ambiance, (day)light and views, bringing together the state-of-the-art EBD knowledge and experience available at the time (2004); specific EBD measures were translated into technical requirements and used to quality-assure each design phase, both for the project as a whole and for the ward environment in particular. We also compared our design measures with the Fable Hospital 2.0 recommendations, and found that many were already in place (Sadler et al., 2011). Creating a safe, pleasant and sustainable built environment was the overarching aim, although decisions were sometimes the result of trade-offs between stakeholder interests, as we reconstructed through evaluation research on this hospital (van Heel et al., 2024).

The biophilic artwork in the corridors and lounge areas was commissioned from a single specialist photographer, ensuring coherence in the murals across outpatient departments, diagnostic areas, operating rooms, and wards (see Photo 2). The architect designed the curtains, drawing inspiration from the colors used in the interior design elements and from the Rotterdam skyline (see Photo 3).

The orchard theme seen in the artwork of photo 2 directly relates to the adjacent accessible roof garden, which features wild apple, pear, and walnut trees. Patients (including those in beds), visitors, and staff make use of this garden, which also offers a spectacular view of the Rotterdam skyline.

The soft chair and table, as well as the rooming-in sofa bed, were originally designed for this project, complementing the soothing color scheme of the SPR and the wider ward environment (see Photo 3).

References:

Korteland, T., Li, C. Y., Dohmen, N., Urbanus, B. H. A., Van Zelst, S., Pu, L., Van Dijk, M., & Ista, E. (2025). The Impact of Hospital Design on Time Spent on Nursing Tasks: A Time Motion Study. *HERD*, *18*(3), 114–124. https://doi.org/10.1177/19375867251330838

Sadler, B. L., Berry, L. L., Guenther, R., Hamilton, D. K., Hessler, F. A., Merritt, C., & Parker, D. (2011). Fable hospital 2.0: the business case for building better health care facilities. *The Hastings Center report*, *41*(1), 13–23. https://doi.org/10.1002/j.1552-146x.2011.tb00093.x

Photo 1: (supplementary file 1d)

*Illustration of the centralized on-stage ward station, with daylight, artwork, and supply trolley; photo by Levien Willemse on behalf of Erasmus MC, with explicit consent of the nurse, 2018*

Photo 2: (supplementary file 1e)

*Illustration of curated artwork in family room in ward environment, photo by Rob van Esch on behalf of Erasmus MC and EGM architects, 2018*

Photo 3: (supplementary file 1f)

*Illustration of an in-use patient room, photo by Liesbeth van Heel on behalf of Erasmus MC with explicit consent of the patient and his wife, 2025*
