# Supplementary figures and images for: Patients’ Experiences of the Transition to a 100% Single-Occupancy Patient Room Hospital in the Netherlands
Source: HERD. 2025 Oct 23;19(1):184–98. doi: 10.1177/19375867251381253 (PMC12715026; doi:10.1177/19375867251381253)

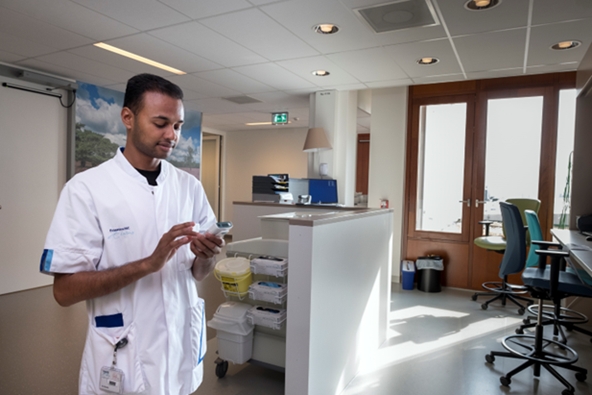

Supplement: sj-jpg-2-her-10.1177_19375867251381253 - Supplemental material for Patients’ Experiences of the Transition to a 100% Single-Occupancy Patient Room Hospital in the Netherlands [file sj-jpg-2-her-10.1177_19375867251381253.jpg]

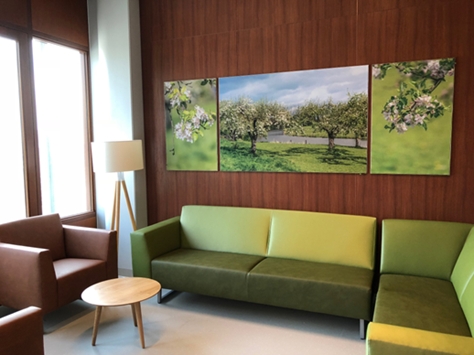

Supplement: sj-jpg-3-her-10.1177_19375867251381253 - Supplemental material for Patients’ Experiences of the Transition to a 100% Single-Occupancy Patient Room Hospital in the Netherlands [file sj-jpg-3-her-10.1177_19375867251381253.jpg]

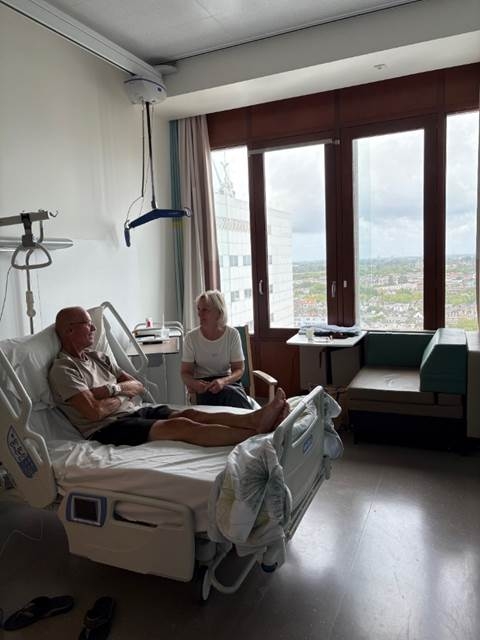

Supplement: sj-jpg-4-her-10.1177_19375867251381253 - Supplemental material for Patients’ Experiences of the Transition to a 100% Single-Occupancy Patient Room Hospital in the Netherlands [file sj-jpg-4-her-10.1177_19375867251381253.jpg]

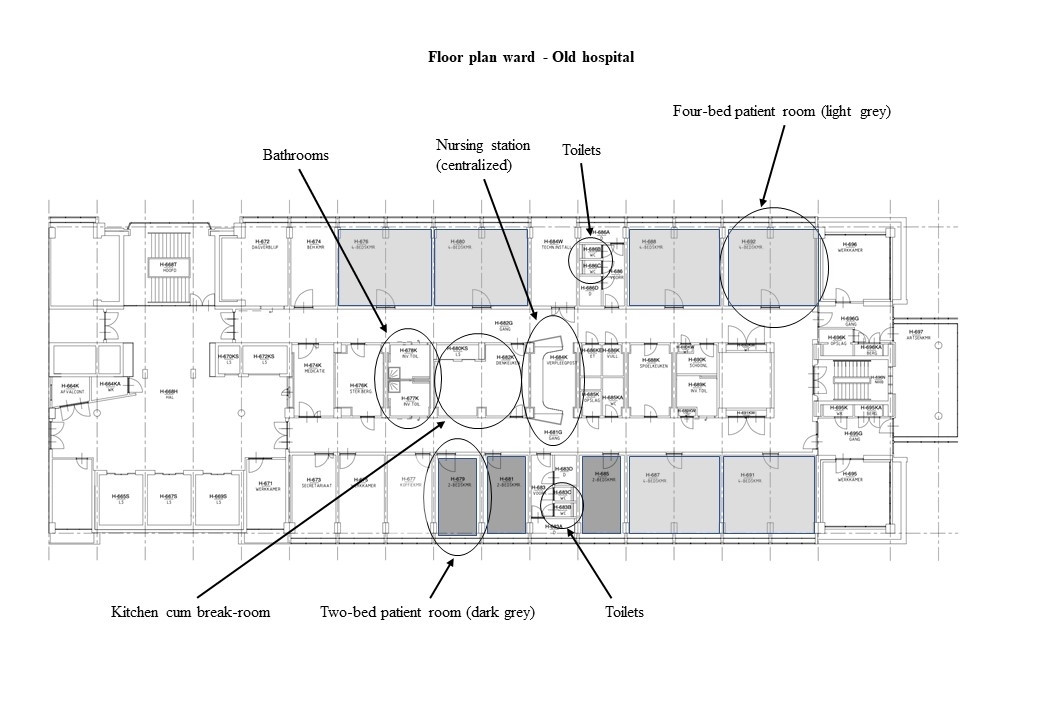

Supplement: sj-jpg-5-her-10.1177_19375867251381253 - Supplemental material for Patients’ Experiences of the Transition to a 100% Single-Occupancy Patient Room Hospital in the Netherlands [file sj-jpg-5-her-10.1177_19375867251381253.jpg]

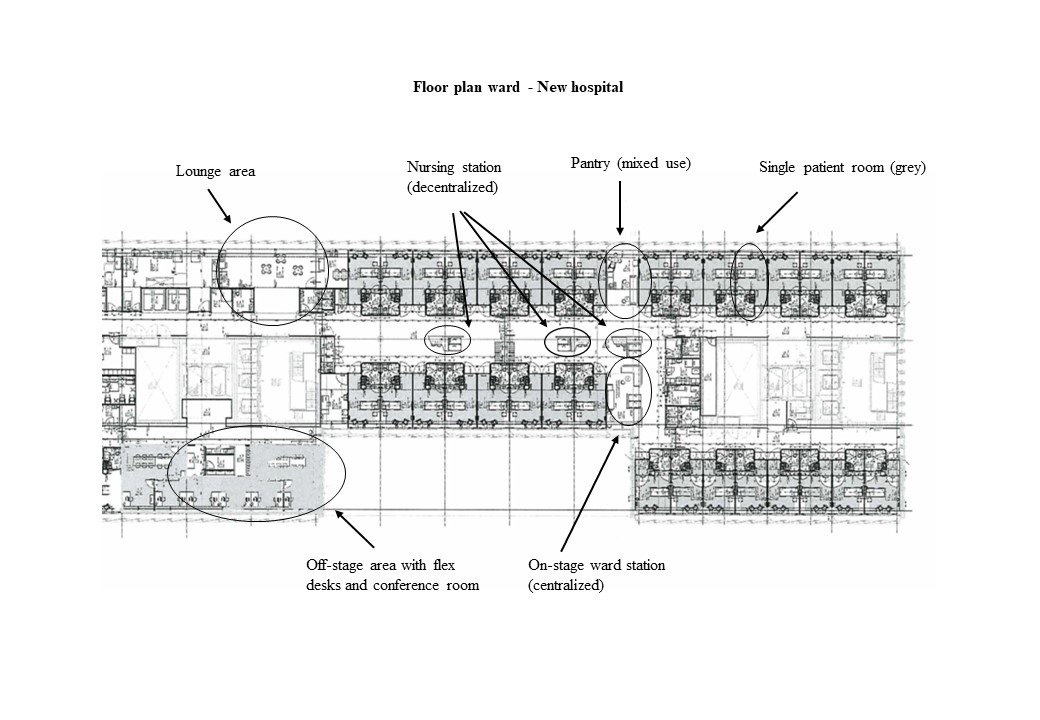

Supplement: sj-jpg-6-her-10.1177_19375867251381253 - Supplemental material for Patients’ Experiences of the Transition to a 100% Single-Occupancy Patient Room Hospital in the Netherlands [file sj-jpg-6-her-10.1177_19375867251381253.jpg]
