# Supplementary material for: Patients’ Experiences of the Transition to a 100% Single-Occupancy Patient Room Hospital in the Netherlands
Source: HERD. 2025 Oct 23;19(1):184–98. doi: 10.1177/19375867251381253 (PMC12715026; doi:10.1177/19375867251381253)
Supplement: sj-docx-7-her-10.1177_19375867251381253 - Supplemental material for Patients’ Experiences of the Transition to a 100% Single-Occupancy Patient Room Hospital in the Netherlands [file sj-docx-7-her-10.1177_19375867251381253.docx]

# WARD ENVIRONMENT QUESTIONNAIRE

## PATIENT DETAILS

| Date of completion: | ____________ | Patient ID number: | ____________ |
| --- | --- | --- | --- |
| Room number: | ____________ | Ward: | ____________ |
| How many patients are in your room (including yourself)? □ 1 □ 2 □ 3 □ 4 | | Patient in isolation: □ Yes □ No | |
|  | | | |

## SOCIO‑DEMOGRAPHICS

- 1. Highest level of education completed: □ University (academic) □ Higher professional □ Secondary □ Lower vocational □ Primary
- 2. Occupation: _________________________ Retired □ Yes □ No Unemployed □ Yes □ No
- 3. Country of birth: ____________________
- 4. Father's country of birth: ____________________
- 5. Mother's country of birth: ____________________
- 6. Language you speak at home most often: ____________________
- 7. Language you speak best: ____________________
- 8. Number of hospital admissions in the past 12 months (including this one): ____

Please indicate how much you agree or disagree with the following statements about the design and facilities of your room and the ward.

Scale: **1** = Strongly disagree **2** = Disagree **3** = Neither agree nor disagree

**4** = Agree **5** = Strongly agree

## ROOM DESIGN

| No. | Item | 1 | 2 | 3 | 4 | 5 |
| --- | --- | --- | --- | --- | --- | --- |
| 1 | My room has a pleasant atmosphere. |  |  |  |  |  |
| 2 | It is important to me to have a tv in my room. |  |  |  |  |  |
| 3 | It is important to me to have a clock in my room. |  |  |  |  |  |
| 4 | It is important to me to have access to internet in my room. |  |  |  |  |  |
| 5 | It is important to me to have art on the wall. |  |  |  |  |  |
| 6 | The view out of my room is appealing |  |  |  |  |  |
| 7 | It is important to me to be able to hang cards or drawings on the wall. |  |  |  |  |  |
| 8 | There is enough space around the bed for visitors. |  |  |  |  |  |
| 9 | There is enough storage space in my room |  |  |  |  |  |
| 10 | I’m able to store my valuable items in a sealed cabinet. |  |  |  |  |  |
| 11 | There is enough space in the room for my family to be able to help me. |  |  |  |  |  |
| 12 | I like my door open during the day. |  |  |  |  |  |
| 13 | I like my door open during the night. |  |  |  |  |  |
| 14 | The lighting around my bed is easy to set |  |  |  |  |  |
| 15 | The temperature in my room is pleasant. |  |  |  |  |  |
| 16 | The remote of the bed is easy to use |  |  |  |  |  |
| 17 | The mattress of my bed is comfortable. |  |  |  |  |  |
| 18 | The pillow is comfortable. |  |  |  |  |  |

## SANITARY FACILITIES

| No. | Item | 1 | 2 | 3 | 4 | 5 |
| --- | --- | --- | --- | --- | --- | --- |
| 19 | The toilet is big enough. |  |  |  |  |  |
| 20 | The toilet is clean enough. |  |  |  |  |  |
| 21 | The toilet is easily accessible (e.g., with infusion stand). |  |  |  |  |  |
| 22 | The bathroom is big enough. |  |  |  |  |  |
| 23 | The bathroom is clean enough. |  |  |  |  |  |
| 24 | The bathroom is easily accessible (e.g., with infusion stand). |  |  |  |  |  |

## WARD LAY-OUT

| No. | Item | 1 | 2 | 3 | 4 | 5 |
| --- | --- | --- | --- | --- | --- | --- |
| 25 | I am able to navigate with ease around the ward. |  |  |  |  |  |
| 26 | I feel like my belongings are safe on the ward. |  |  |  |  |  |
| 27 | There are plenty of places outside the room to sit with visitors. |  |  |  |  |  |
| 28 | The ward has plenty of space to walk around. |  |  |  |  |  |
| 29 | Ambient noises (e.g. phones, squeaky doors, equipment) disturb me during the day. |  |  |  |  |  |
| 30 | Ambient noises (e.g. phones, squeaky doors, equipment) disturb me during the night. |  |  |  |  |  |
| 31 | If I like, I can interact with other patients. |  |  |  |  |  |
| 32 | The day-care room and seating areas on the ward are pleasant. |  |  |  |  |  |
| 33 | **Only in the new hospital building:**  I do miss a fellow patient to talk to. |  |  |  |  |  |

## PRIVACY

| No. | Item | 1 | 2 | 3 | 4 | 5 |
| --- | --- | --- | --- | --- | --- | --- |
| 34 | I experience sufficient privacy during doctors’ visit. |  |  |  |  |  |
| 35 | I experience sufficient privacy during physical examination or care in bed. |  |  |  |  |  |
| 36 | I experience sufficient privacy in my room during a one‑on‑one conversation with my physician or nurse. |  |  |  |  |  |
| 37 | I experience sufficient privacy when my relatives and friends come to visit me. |  |  |  |  |  |
| 38 | The toilet offers sufficient privacy. |  |  |  |  |  |
| 39 | The bathroom offers sufficient privacy. |  |  |  |  |  |
| 40 | I experience sufficient privacy when I need to use a bedpan. |  |  |  |  |  |

## OPEN QUESTIONS

1. What do you think about having family stay overnight in your room?

____________________________________________________________________

2. What advantages do you see in a two‑ or four‑bed room?

____________________________________________________________________

3. What advantages do you see in a single‑bed room?

____________________________________________________________________

4. If you could change one thing to make your stay more pleasant, what would it be?

____________________________________________________________________

Thank you for completing this questionnaire.
