# Supplementary material for: Patients’ Experiences of the Transition to a 100% Single-Occupancy Patient Room Hospital in the Netherlands
Source: HERD. 2025 Oct 23;19(1):184–98. doi: 10.1177/19375867251381253 (PMC12715026; doi:10.1177/19375867251381253)
Supplement: sj-docx-8-her-10.1177_19375867251381253 - Supplemental material for Patients’ Experiences of the Transition to a 100% Single-Occupancy Patient Room Hospital in the Netherlands [file sj-docx-8-her-10.1177_19375867251381253.docx]

**Supplementary File** **3: Privacy**

Detailed responses of participants to statements concerning privacy, in the former and new hospital buildings

|  | **Former hospital building**  **N (%)** | | | **New hospital building**  **N (%)** | | | **p-value*** |  |
| --- | --- | --- | --- | --- | --- | --- | --- | --- |
|  | (Totally) disagree | Not disagree, not agree | (Totally) agree | (Totally) disagree | Not disagree, not agree | (Totally) agree |  |  |
| I experience sufficient privacy during doctors’ visit | 54 (24.9) | 31 (14.3) | 132 (60.8) | 8 (2.1) | 6 (1.6) | 371 (96.4) | **<0.001** |  |
| I experience sufficient privacy during physical examination or care in bed | 23 (10.6) | 24 (11.1) | 170 (78.3) | 7 (1.9) | 6 (1.6) | 364 (96.6) | **<0.001** |  |
| I experience sufficient privacy in my room during a one-on-one conversation with my physician or nurse | 67 (31.2) | 26 (12.1) | 122 (56.7) | 11 (2.8) | 5 (1.3) | 378 (95.9) | **<0.001** |  |
| I experience sufficient privacy when my relatives and friends come to visit me | 43 (20.7) | 32 (15.4) | 133 (63.9) | 3 (0.8) | 3 (0.8) | 391 (98.5) | **<0.001** |  |
| The toilet offers sufficient privacy | 11 (5.3) | 19 (9.1) | 179 (85.6) | 2 (0.5) | 0 (-) | 403 (99.5) | **<0.001** |  |
| The bathroom offers sufficient privacy | 9 (4.4) | 19 (9.3) | 176 (86.3) | 2 (0.5) | 0 (-) | 403 (99.5) | **<0.001** |  |
| I experience sufficient privacy when I need to use a bedpan | 37 (28.7) | 15 (11.6) | 77 (59.7) | 7 (3.8) | 9 (4.9) | 167 (91.3) | **<0.001** |  |
| The option ‘not applicable’ is handled as missing value.  * Chi-square analyses, significant if p <0.05 | | | | | | | | |
