# Supplementary material for: Patients’ Experiences of the Transition to a 100% Single-Occupancy Patient Room Hospital in the Netherlands
Source: HERD. 2025 Oct 23;19(1):184–98. doi: 10.1177/19375867251381253 (PMC12715026; doi:10.1177/19375867251381253)
Supplement: sj-docx-9-her-10.1177_19375867251381253 - Supplemental material for Patients’ Experiences of the Transition to a 100% Single-Occupancy Patient Room Hospital in the Netherlands [file sj-docx-9-her-10.1177_19375867251381253.docx]

**Supplementary File 4: Sanitary facilities**

Detailed responses of participants to statements concerning sanitary facilities, in the former and new hospital buildings

|  | **Former hospital bulding**  **N (%)** | | | **New hospital building**  **N (%)** | | | **p-value*** |  |
| --- | --- | --- | --- | --- | --- | --- | --- | --- |
|  | (Totally) disagree | Not disagree, not agree | (Totally) agree | (Totally) disagree | Not disagree, not agree | (Totally) agree |  |  |
| The toilet is big enough | 41 (19.5) | 15 (7.1) | 154 (73.3) | 3 (0.7) | 2 (0.5) | 399 (98.8) | **<0.001** |  |
| The toilet is clean enough | 43 (20.6) | 25 (12.0) | 141 (67.5) | 16 (4.0) | 16 (34.0) | 366 (92.0) | **<0.001** |  |
| The toilet is easily accessible  (e.g. with infusion stand) | 34 (16.7) | 28 (13.7) | 142 (69.6) | 6 (1.6) | 7 (1.9) | 362 (96.5) | **<0.001** |  |
| The bathroom is big enough | 30 (14.9) | 15 (7.4) | 157 (77.7) | 6 (1.5) | 4 (1.0) | 393 (97.5) | **<0.001** |  |
| The bathroom is clean enough | 34 (17.0) | 23 (11.5) | 143 (71.5) | 17 (4.3) | 15 (3.8) | 366 (92.0) | **<0.001** |  |
| The bathroom is easily accessible (e.g. with infusion stand) | 25 (12.7) | 22 (11.2) | 150 (76.1) | 8 (2.2) | 9 (2.4) | 354 (95.4) | **<0.001** |  |
| The option ‘not applicable’ is handled as missing value.  * Chi-Square analyses, significant if p <0.05 | | | | | | | | |
