# Supplementary material for: Patients’ Experiences of the Transition to a 100% Single-Occupancy Patient Room Hospital in the Netherlands
Source: HERD. 2025 Oct 23;19(1):184–98. doi: 10.1177/19375867251381253 (PMC12715026; doi:10.1177/19375867251381253)
Supplement: sj-docx-10-her-10.1177_19375867251381253 - Supplemental material for Patients’ Experiences of the Transition to a 100% Single-Occupancy Patient Room Hospital in the Netherlands [file sj-docx-10-her-10.1177_19375867251381253.docx]

**Supplementary File 5: Room design**

Detailed responses of participants to statements concerning room design, in the former and new hospital buildings

| **Question** | **Former hospital building, n (%)** | | | **New hospital building, n (%)** | | | **P-value*** |
| --- | --- | --- | --- | --- | --- | --- | --- |
|  | (Totally) disagree | Not disagree, not agree | (Totally) agree | (Totally) disagree | Not disagree, not agree | (Totally) agree |  |
| My room has a pleasant atmosphere | 96 (42.7) | 35 (15.6) | 94 (41.8) | 7 (1.7) | 13 (3.1) | 393 (95.2) | **<0.001** |
| It is important to me to have a tv in my room | 60 (27.6) | 23 (10.6) | 134 (61.8) | 35 (8.4) | 26 (6.3) | 354 (85.3) | **<0.001** |
| It is important to me to have a clock in my room | 25 (11.1) | 10 (4.4) | 191 (84.5) | 32 (7.7) | 34 (8.2) | 347 (84.0) | **<0.001** |
| It is important to me to have access to internet in my room | 39 (17.8) | 15 (6.8) | 165 (75.3) | 87 (21.2) | 34 (8.3) | 289 (70.5) | **<0.001** |
| It is important to me to have art on the wall | 80 (37.2) | 52 (24.2) | 83 (38.6) | 178 (43.2) | 109 (26.5) | 125 (30.3) | **0.001** |
| The view out of my room is appealing | 77 (34.7) | 23 (10.4) | 122 (55.0) | 94 (23.0) | 44 (10.7) | 274 (66.3) | **<0.001** |
| It is important to me to be able to hang cards or drawings on the wall | 37 (16.8) | 29 (13.2) | 154 (70.0) | 96 (23.3) | 62 (15.0) | 254 (61.7) | **<0.001** |
| There is enough space around the bed for visitors | 89 (40.8) | 13 (6.0) | 154 (53.2) | 0 (-) | 8 (1.9) | 406 (98.1) | **<0.001** |
| There is enough storage space in my room | 57 (25.6) | 21 (9.4) | 145 (65.0) | 66 (16.0) | 42 (10.2) | 304 (73.8) | **<0.001** |
| I’m able to store my valuable items in a sealed cabinet | 34 (15.4) | 16 (7.2) | 171 (77.4) | 27 (6.7) | 19 (4.7) | 355 (88.5) | **<0.001** |
| There is enough space in the room for my family to be able to help me | 61 (29.2) | 34 (16.3) | 144 (54.5) | 1 (0.3) | 12 (3.1) | 379 (96.7) | **<0.001** |
| I like my door to be open during the day | 17 (9.2) | 19 (10.3) | 149 (80.5) | 65 (16.0) | 70 (17.2) | 272 (66.8) | **<0.001** |
| I like my door to be open during the night | 74 (39.8) | 25 (13.4) | 87 (46.8) | 241 (59.7) | 47 (11.6) | 116 (28.7) | **<0.001** |
| The lighting around my bed is easy to set | 41 (19.1) | 22 (10.2) | 152 (70.7) | 112 (28.4) | 34 (8.6) | 249 (63.0) | **<0.001** |
| The temperature in my room is pleasant | 55 (24.4) | 30 (13.3) | 140 (62.2) | 49 (11.8) | 38 (9.2) | 327 (79.0) | **<0.001** |
| The remote of the bed is easy to use | 17 (7.6) | 20 (9.0) | 186 (83.4) | 19 (4.7) | 10 (2.5) | 379 (92.9) | **0.001** |
| The option ‘not applicable’ is handled as missing value.  * Chi-square analyses, significant if p <0.05 | | | | | | | |
