# Supplementary material for: Patients’ Experiences of the Transition to a 100% Single-Occupancy Patient Room Hospital in the Netherlands
Source: HERD. 2025 Oct 23;19(1):184–98. doi: 10.1177/19375867251381253 (PMC12715026; doi:10.1177/19375867251381253)
Supplement: sj-docx-11-her-10.1177_19375867251381253 - Supplemental material for Patients’ Experiences of the Transition to a 100% Single-Occupancy Patient Room Hospital in the Netherlands [file sj-docx-11-her-10.1177_19375867251381253.docx]

**Supplementary File 6: Ward lay-out**

Detailed responses of participants to statements concerning ward lay-out, in the former and new hospital buildings

|  | **Former hospital building**  **N (%)** | | | **New hospital building**  **N (%)** | | | **p-value*** |
| --- | --- | --- | --- | --- | --- | --- | --- |
|  | (Totally) Disagree | Not disagree, not agree | (Totally) Agree | (Totally) disagree | Not disagree,  Not agree | (Totally) agree |  |
| I am able to navigate with ease around the ward | 20 (10.8) | 19 (10.3) | 146 (78.9) | 39 (13.5) | 41 (14.2) | 208 (72.2) | **0.004** |
| I feel like my belongings are safe on the ward | 19 (8.8) | 32 (14.9) | 164 (76.3) | 15 (3.8) | 35 (8.8) | 350 (87.5) | **0.003** |
| There are plenty of places outside the room to sit with visitors | 46 (27.9) | 40 (24.2) | 79 (47.9) | 11 (4.7) | 19 (8.1) | 206 (87.3) | **<0.001** |
| The ward has plenty of space to walk around | 28 (15.9) | 25 (14.2) | 123 (69.9) | 9 (3.2) | 19 (6.7) | 254 (90.1) | **<0.001** |
| Ambient noises (e.g. phones, squeaky doors, equipment) disturb me during the day | 118 (54.1) | 44 (20.2) | 56 (25.7) | 321 (79.1) | 34 (8.4) | 49 (12.1) | **<0.001** |
| Ambient noises (e.g. phones, squeaky doors, equipment) disturb me during the night | 98 (46.0) | 21 (9.9) | 94 (44.1) | 317 (82.8) | 20 (5.2) | 46 (12.0) | **<0.001** |
| If I like, I can interact with other patients | 14 (8.4) | 26 (15.7) | 126 (75.8) | 77 (28.3) | 51 (18.8) | 144 (52.9) | **<0.001** |
| The day-care room and seating areas on the ward are pleasant | 44 (28.4) | 47 (30.3) | 64 (41.3) | 5 (2.5) | 22 (10.9) | 175 (86.6) | **<0.001** |
| The option ‘not applicable’ is handled as missing value.  * Chi-square analyses, significant if p <0.05 | | | | | | | |
